# Enlarged Prostate

**Published:** 1872-06

**Authors:** Geo. F. Taylor

**Affiliations:** Alabama


### ENLARGED PROSTATE.

BY GEO. F. TAYLOR, M. D., OF ALABAMA.

The following, though an unsuccessful case, may not be uninteresting to the readers of THE COMPANION; while some may be benefitted by the suggestions of the special practice; the facts may elicit a thought from others, that may be of service, not only to the communicant but perhaps may add something to the literature of medicine.

Mr. N—, æt. 75, was taken on the evening of April 23d, 1872, with suppression of urine—during the day, he had not micturated, but felt but little inconvenience until nightfall, at which time the desire to micturate had increased to an extent that gave distress.

He was of ruddy complexion, and as far as he knew, was of vigorous health, and nothing but the suppression seemed to indicate any disorder. By midnight, the distress had amounted to an agony. In this condition he remained until 6 o'clock of the 24th, when he was visited by my friend, Dr. B. F. Rea, who, on an exploration, found the pelvis filled by an enlarged prostate, which bore against the perineum and rectum, and seemed to lift the distended bladder upward into the abdomen. Every effort at the introduction of the catheter failed, and disclosed the fact of a stricture in the prostatic urethra. At 11 o'clock of the 24th, I saw him (being called to assist in the case).

I found the prostate as above described—complexion was ruddy—tongue slightly touched with a white fur, pulse full and about eighty per minute—urine mixed with blood, was constantly dripping from the urethra. Another effort was now made at the introduction of the catheter—different sized instruments were used with a change of position of patient, also venesection was used while the catheter was pressed in—also, the hot bath followed by the instrument; lastly, belladonna upon the perineum hyperdermically, and also per urethram. The instrument would reach the prostate and seem to enter some points of the prostate stricture, but the bladder could not be entered, and an introduction in that way was finally abandoned. What then was to be done? A supersymphysis puncture would only give temporary relief, and for the balance of time would make an unnatural and dangerous outlet, for without relief at the urethra, all openings, whether through the rectum or above the symphysis, would necessarily remain fistulous, with a constant tendency to infiltration. Discovering small discharges of urine after each bathing (amounting, sometimes, to an ounce), as also the constant stillicidium, induced us to follow the indication, with a hope of ultimate success at relief, in aid of which he was well purged (each stool being attended with considerable hemorrhages), and again and again, put into the hot bath, each time being followed by the usual discharge.

This practice was followed for forty-eight hours, with occasional urethal injections of tincture belladonna.

Dispairing of full success by this course, we decided to cut for the stricture, although it might implicate the prostate in the incision. Placing the patient upon a table in the position for lithotomy, with a large catheter pushed down upon the stricture, then giving chloroform, the operation was commenced. The skin of the perineum being laid open, left bare the enlarged and hardened prostate. I made the vertical cut and pushed the scalpel along up and through the prostate, until I reached the membranous urethra, this I laid open and slowly pushed the scalpel along the tract of the catheter, until I reached a semi-cartilaginous formation in advance of the catheter, and on the neck of the bladder. This being cut the catheter readily slipped into the bladder, and the urine was thus drawn off—no great amount of hemorrhage ensued, and about half a gallon of water was taken away.

The cut disclosed the fact that, the enlarged prostate filled the pelvis, as before suggested. It was spongy to the feel, and offered to the knife about as much resistance as ordinary cheese. The catheter was left in, and the wound stitched. There was never any percolation through the cut, but the urine passed per catheter; his pulse recovered its strength—distress was relieved, and his skin was moist; some stimulants and a gentle tonic were given, with fair chances for success, for thirty-six hours; after this, slight rigors came on, collapse ensued, and he expired in about forty-eight hours after the operation. I would here remark, that soon after the operation, his bowels moved, the stool was bilious and mucous, with considerable hemorrhage.

Was there not previous prostatic enlargement? Could it have been relieved by any plan of medication, provided the supersymphisis operation had been performed at an early hour? Would any course have relieved this case? I would be glad to see the opinions of the profession in relation thereto.

